# The Impact of Population Variation in the Analysis of microRNA Target Sites

**DOI:** 10.3390/ncrna5020042

**Published:** 2019-06-22

**Authors:** Mohab Helmy, Andrea Hatlen, Antonio Marco

**Affiliations:** School of Biological Sciences, University of Essex, Colchester CO4 3SQ, UK; mhaelb@essex.ac.uk (M.H.); a.hatlen@essex.ac.uk (A.H.)

**Keywords:** miRNAs, human populations, gene regulation, microRNA target prediction, evolution

## Abstract

The impact of population variation in the analysis of regulatory interactions is an underdeveloped area. MicroRNA target recognition occurs via pairwise complementarity. Consequently, a number of computational prediction tools have been developed to identify potential target sites that can be further validated experimentally. However, as microRNA target predictions are done mostly considering a reference genome sequence, target sites showing variation among populations are neglected. Here, we studied the variation at microRNA target sites in human populations and quantified their impact in microRNA target prediction. We found that African populations carry a significant number of potential microRNA target sites that are not detectable in the current human reference genome sequence. Some of these targets are conserved in primates and only lost in Out-of-Africa populations. Indeed, we identified experimentally validated microRNA/transcript interactions that are not detected in standard microRNA target prediction programs, yet they have segregating target alleles abundant in non-European populations. In conclusion, we show that ignoring population diversity may leave out regulatory elements essential to understand disease and gene expression, particularly neglecting populations of African origin.

## 1. Introduction

The study of gene regulatory iterations is at the heart of biomedical research. Many disease-causing mutations affect regulatory motifs that eventually lead to a miss-regulation of fine-tuned biological processes. In the last years, microRNAs have emerged as an important type of regulatory molecules involved in virtually all biological networks [1,2]. Both the deletion and overexpression of microRNAs have been associated to human diseases (see for instance the compilation by [3]). Notably, the role of microRNAs in cancer is a particularly active research field [4,5]. Unlike other regulatory molecules (such as transcription factors or RNA binding proteins), the interaction between regulator and target is mediated by pairwise nucleotide complementary [1]. Specifically, a microRNA is partially complementary to motifs in RNA transcripts and, after binding, translational repression or even RNA degradation mechanisms are triggered [1,6]. Indeed, changes at microRNA target sites are now associated to major human diseases including cancer, neurodegeneration and metabolic disorders (see [7,8] and references therein), among others. The study of microRNA target sites is becoming more important as we start to understand the complexities behind microRNA regulatory networks.

From a computational point of view, the microRNA targeting mechanisms have a clear advantage: targets can be predicted by scanning genomic sequences for potential complementary sites [9,10,11]. However, microRNA target prediction algorithms have, in general, high rates of false positives [12,13]. Consequently, most research on microRNA targets complements a computational sieve of potential target sites plus experimental validations, usually by luciferase assays or similar (e.g., [14]). Therefore, the first step in microRNA target analysis is the prediction of binding sites from a primary sequence. In biomedical research, this primary sequence is often, if not always, the human reference genome sequence (currently hg38). However, a reference genome does not take into account existing variation within human populations and, therefore, any inference from such a sequence may be biased. Populations of African origin show a higher nucleotide diversity than other populations, probably as a consequence of a recent origin of Eurasian populations from a small group of African migrants (reviewed in [15]). There exist several projects aimed to account for all this nucleotide variability in human populations (i.e., [16]). Nevertheless, microRNA target prediction programs rely on reference genome sequences (as other regulatory interaction programs do), where this diversity is not truly represented.

Variation at gene regulatory sites, particularly at transcription factor binding sites, has been studied in the past (e.g., [17,18]). In microRNA target sites, some studies have shown that, as expected, functional target sites are often conserved in populations [19,20]. On the other hand, the creation of novel, potentially harmful, microRNA target sites, is selected against in populations [21,22]. In summary, the literature clearly shows evidence that nucleotide variation occurs at microRNA target sites. The question is, how much of this variation is neglected in the human reference genome sequence, and therefore not accounted for in standard microRNA target prediction programs? In other words, what is the impact of nucleotide variation among humans in microRNA target prediction research? This is the question we are tackling in this paper.

## 2. Results and Discussion

**Figure 1:**
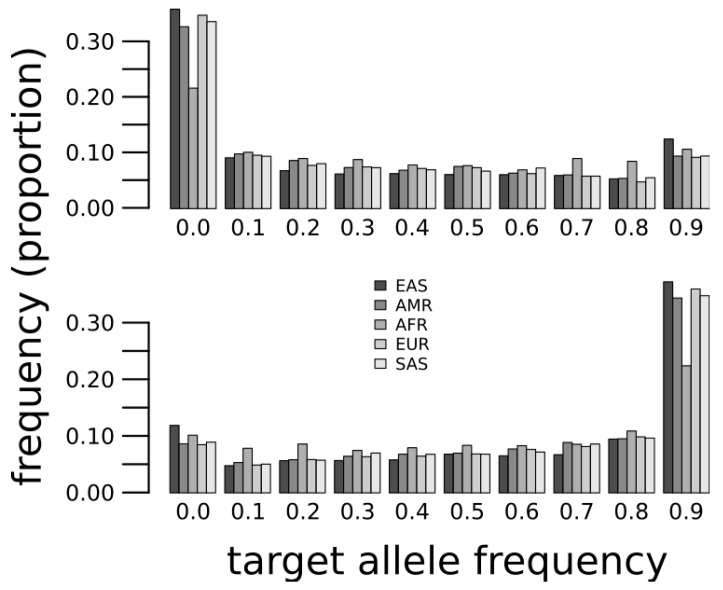
Target allele frequencies at microRNA target sites where the target is ancestral and not in the reference genome sequence (**top**) or the target is derived and present in the reference genome sequence (**bottom**). Super-populations are African (AFR), Mixed American (AMR), East Asian (EAS), European (EUR) and South Asian (SAS).

We compiled biallelic SNPs at predicted microRNA target sites (see Methods) for which one of the alleles is a target site and the other is not a target site. These are the target/near-target pairs as defined before [11,21]. Considering that populations of African origin host more variation, including ancestral alleles, than other populations, we hypothesize that ancestral target sites that are not present in the reference genome sequence were lost in Out-of-Africa populations. Thus, we first consider target sites whose allele in the reference genome sequence is not a target, yet the ancestral allele was a target site. The distribution of these alleles frequencies in five major groups (African, European, South Asian, Native American and East Asian) indicates that non-African populations tend to have the non-target allele whilst African populations show a wider variation, and a paucity of non-targets compared to the other populations (Figure 1, top). Additionally, we consider target sites in the reference genome sequence that were not ancestral to human populations and, as expected, we found that African populations have lower frequencies of the target allele (Figure 1, bottom). These results suggest that a significant loss and gain of microRNA target sites happened in Out-of-African populations.

To illustrate the impact of variation at target sites we selected SNPs with a very high degree of population differentiation (Fst > 0.7; see Methods) that are not present in the human genome reference sequence. We found 26 target sites with such a degree of variation across populations (Table 1) for highly expressed microRNAs. Many of these target sites are lost in Out-of-Africa populations, suggesting that losses rather than the gains of target sites are more likely to happen during differentiation between populations (see discussion in [22]).

**Figure 2:**
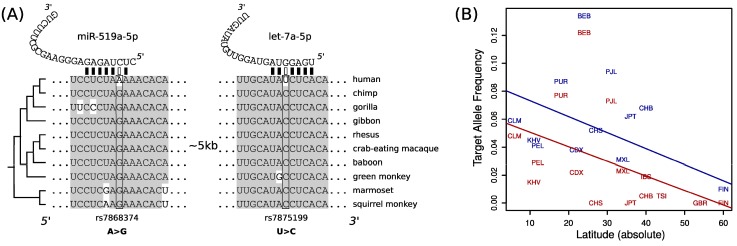
MicroRNA target sites variation at transcript from the MTAP gene: (**A**) alignment of 3′UTR fragments from 10 primate species, including human, and the location of the target sites and the polymorphic nucleotides; (**B**) scatter-plot between the latitude (in absolute value) and the target allele frequency for target sites for miR-519a-5p (red) and let-7a-5p (blue). Straight lines are fitted linear models (see main text).

We observed that *MTAP* have lost two target sites for two highly expressed microRNAs in non-African populations. These two target sites were the ancestral form in human populations and conserved as targets in other primates (Figure 2A). Genome-wide association studies have linked variants at *MTAP* with the abundance of naevi (moles) and also with melanoma incidence [23,24], although the association between *MTAP* and melanoma seems to be rather complex [23]. We speculate that the loss of two target sites at MTAP transcripts could be associated with an elevated expression of the gene in Out-of-Africa populations, perhaps by a relaxation on the selective pressures that maintained the target sites under purifying selection in African populations, where light exposure is higher, and an overproduction of MTAP may be linked to a higher incidence of skin cancer. Under this hypothesis, we expect the target allele frequency to be also negatively correlated with the latitude of sampling of non-African human populations. We compiled sampling information for human populations (see Methods) and build a linear model to predict the target allele frequency as a function of the latitude. We observed that populations from higher latitudes have lower target allele frequencies at both of the studied target sites (Figure 2B), although the statistical support was weak (rs7875199: R^2^ (adjusted) = 0.18, *p* = 0.06; rs7868374: R^2^ (adjusted) = 0.14, *p* = 0.09). We would like to emphasize that the association between latitude and MTAP target site allele frequencies is still speculative, but the fact that other similar associations has been found at microRNA target sites (e.g., [25]) suggest that other latitude depending microRNA function remain to be discovered.

Likewise, we found a similar pattern in two target sites at *CYB5R4*, a gene associated to gene-environment interactions [26,27]. In particular, *CYB5R4* has been associated to the risk of diabetes, and variants at its 5′ region have been found to be specific of African populations [26]. The association between population variants and disease-related genes such as *MTAP* and *CYB5R4* remains highly speculative, yet it is very suggestive. In any case, the identified target sites at *MTAP* and *CYB5R4* are not present in the human genome reference genome yet they are highly abundant in African populations and conserved in primates, indicating that target prediction programs often neglect an important part of human variability.

We identified microRNA/gene interactions that have been experimentally detected in high-throughput experiments with a significant population differentiation (P_Fst_ < 0.05, see Methods) that were not detected as targets in the reference genome sequence (Table 2). For instance, miR-24-3p, a tumour-suppressor microRNA [28], has a polymorphic target site in Caspase 10 transcripts, whose product is an component of the apoptotic machinery. This target site is present in about 26% or African sampled alleles whilst it is not detected in most other populations. Indeed, neither TargetScan nor DIANA-microT [10] predict this interaction. This result further supports the view that reference genome sequences used to predict microRNA target sites often ignore population variability that may indicate functional diversity.

**Figure 3:**
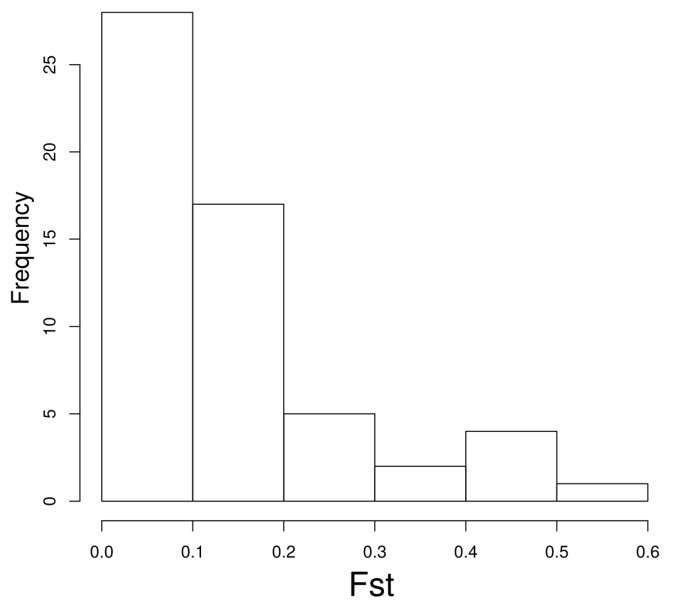
Fst values for polymorphic microRNA target sites experimentally evaluated with PASSPORT-seq (see main text).

As this work is a computational evaluation of potential microRNA target sites, we have not validated any of the targets. However, we wanted to explore whether experimentally validated polymorphic target sites showed some population differentiation. Hence, we explored the 111 variants tested by PASSPORT-seq [29] of which 59 were listed in our database. The distribution of Fst values shows population differentiation for some targets (Figure 3). For instance, eight target sites (over 13% of the total) had an associated Fst value of 0.3 or more.

Last, we investigated polymorphic target sites identified by the popular software TargetScan. We downloaded the whole conserved and non-conserved target datasets and identified polymorphic target sites also present in our database PopTargs. We compared the target allele frequencies between populations and concluded that, for both conserved and nonconserved targets, the target allele was more common in European than in African populations (Table 3). Indeed, for nonconserved targets, the differences are larger. 

In conclusion, here, we showed that the study of variation in human populations reveals the presence of microRNA target sites that are not detected with current methodological frameworks. We suggest that variation at target sites must be taken into account in biomedical studies as some populations (mostly of African origin) may be neglected. Further research is needed to quantify the impact of population variation in the function of microRNA target sites.

## 3. Methods

Polymorphic sites at predicted microRNA target sites were retrieved from the PopTargs database (version 2; Hatlen, Helmy and Marco, under review; https://poptargs.essex.ac.uk). Only target sites for highly expressed microRNAs were considered. Population frequencies, Fst values and ancestral alleles were also retrieved in PopTargs, as provided in [30]. We only consider segregating alleles with a frequency between 0.01 and 0.99. Sequence alignment of primate sequences was extracted using the UCSC table browser [31]. The geographical location (latitude) of the genome samples sequenced in the 1000 genomes project was obtained from the sample descriptions at https://www.coriell.org/1/NHGRI/Collections/1000-Genomes-Collections/1000-Genomes-Project. When the specific location was ambiguous, we used the location of the capital city of the country of origin. For multiple collection points we computed the centroid middle point. Recently migrated populations were discarded (CHD, CEU, ASW, ACB, GIH, STU and ITU as described at IGSR: The International Genome Sample Resource (http://www.internationalgenome.org/category/population/)) were discarded from the analysis in Figure 2B. We extracted microRNA targets from the miRTarBase database [32], which were detected only in high-throughput experiments (PAR-CLIP and/or HITS-CLIP).

## Figures and Tables

**Table 1:** MicroRNA target sites, not in the reference genome sequence, with a very high differentiation among human populations.

MicroRNA	Gene	SNP ^a^	EAS ^b^	AMR ^b^	AFR ^b^	EUR ^b^	SAS ^b^	Fst
miR-519a-5p ^1^	MTAP	rs7868374	0.0079	0.0476	0.6838	0.0050	0.1176	0.8239
let-7a-5p ^2^	MTAP	rs7875199	0.0536	0.0576	0.7958	0.0070	0.1288	0.7997
miR-337-3p ^3^	ATP1A1	rs1885802	0.0427	0.1268	0.8109	0.0338	0.0450	0.7914
miR-185-3p ^4^	SLC5A10	rs1624825	0.9970	0.5159	0.9667	0.2028	0.7495	0.7859
miR-1180-3p	MYEF2	rs2470102	0.7530	0.3386	0.9251	0.0060	0.2720	0.7726
miR-151a-5p ^5^	SCN2B	rs624328	0.9405	0.8905	0.2519	0.9513	0.9673	0.7674
miR-4732-5p	AP5M1	rs1889720	0.9335	0.9107	0.1551	0.9433	0.9182	0.7637
miR-584-5p	CYB5R4	rs6903739	0.0000	0.0749	0.7738	0.0477	0.0061	0.7514
miR-618	CYB5R4	rs9449733	1.0000	0.9251	0.2247	0.9523	0.9939	0.7397
miR-18a-3p	ANKRD65	rs904589	0.1131	0.1945	0.8222	0.0964	0.1881	0.7332
miR-329-3p ^6^	CEP162	rs6901546	0.0139	0.1628	0.7670	0.0706	0.0900	0.7326
miR-150-5p ^7^	HM13	rs6059873	1.0000	0.8156	0.1725	0.8429	0.9622	0.7298
miR-513a-3p ^8^	TCERG1	rs3822506	0.7183	0.1167	0.0325	0.0915	0.2198	0.7224
miR-128-1-5p ^9^	BCL7C	rs11864054	0.9117	0.5447	0.0242	0.3817	0.1595	0.7144
miR-192-5p ^10^	C12orf65	rs1533703	0.9980	0.7262	0.1490	0.7694	0.7945	0.7074

^a^ SNP names are dbSNP accession numbers. ^b^ Allele frequencies refer to the target allele frequency in each of the five super-population groups. Superscript in microRNA names indicates other mature sequences with the same target site: ^1^ miR-520c-5p, miR-519b-5p, miR-526a-5p, miR-519c-5p, miR-518f-5p, miR-523-5p, miR-518d-5p, miR-522-5p, miR-518e-5p; ^2^ let-7e-5p, let-7c-5p, let-7f-5p, let-7d-5p, let-7b-5p, miR-98-5p, let-7i-5p, let-7g-5p, miR-1294; ^3^ miR-202-5p; ^4^ miR-128-1-5p; ^5^ miR-561-5p, miR-3605-5p, miR-24-3p, miR-151b; ^6^ miR-362-3p; ^7^ miR-296-3p, miR-4446-3p, miR-23b-5p, miR-3158-3p; ^8^ miR-513c-3p; ^9^ miR-491-5p, miR-1296-5p; 10miR-215-5p.

**Table 2:** Target sites identified in high-throughput experiments with high population differentiation.

MicroRNA	Gene	SNP ^a^	EAS ^b^	AMR ^b^	AFR ^b^	EUR ^b^	SAS ^b^	Fst
miR-1307-3p	VSTM4	rs4240499	0.2361	0.4971	0.9017	0.3976	0.5031	0.5352
miR-512-3p	EIF2B2	rs4556	0.8393	0.6441	0.1884	0.5388	0.5194	0.4327
miR-197-3p	DBT	rs6701655	0.9296	0.8905	0.4168	0.8648	0.8978	0.4186
miR-17-5p	MRPS10	rs3199638	0.7937	0.6369	0.2148	0.6372	0.7679	0.3946
miR-326	SLC27A4	rs7048106	0.3185	0.2176	0.7617	0.2386	0.3476	0.3782
miR-24-3p	CASP10	rs13432040	0.000	0.0259	0.2587	0.000	0.000	0.2943
miR-342-3p	LRPAP1	rs3468	0.5089	0.2507	0.1611	0.3489	0.2669	0.238

^a^ SNP names are dbSNP accession numbers. ^b^ Allele frequencies refer to the target allele frequency in each of the five super-population groups.

**Table 3:** Wilcoxon signed-rank (paired) test between African and European target allele frequencies in TargetScan predicted microRNA target sites.

TargetScan Dataset	Sample Size (pairs)	*p*	Shift Median ^1^	Shift 95% CI^2^
conserved	9245	<2.2 × 10^−16^	7.976 × 10^−4^	(7.833–7.878) 10^−4^
nonconserved	1041868	<2.2 × 10^−16^	1.174 × 10^−3^	(1.151–1.210) 10^−3^

^1^ Estimated shift median for the difference: European minus African target allele frequency. ^2^ Confidence Interval.
